# Patient preferences and quality of life implications of ravulizumab (every 8 weeks) and eculizumab (every 2 weeks) for the treatment of paroxysmal nocturnal hemoglobinuria

**DOI:** 10.1371/journal.pone.0237497

**Published:** 2020-09-04

**Authors:** John Devin Peipert, Austin G. Kulasekararaj, Anna Gaya, Saskia M. C. Langemeijer, Susan Yount, F. Ataulfo Gonzalez-Fernandez, Emilio Ojeda Gutierrez, Christa Martens, Amy Sparling, Kimberly A. Webster, David Cella, Ioannis Tomazos, Masayo Ogawa, Caroline I. Piatek, Richard Wells, Flore Sicre de Fontbrune, Alexander Röth, Lindsay Mitchell, Anita Hill, Karen Kaiser

**Affiliations:** 1 Department of Medical Social Sciences, Northwestern University Feinberg School of Medicine, Chicago, IL, United States of America; 2 Department of Haematological Medicine, King’s College Hospital, NIHR/Wellcome King’s Clinical Research Facility, and King’s College London, London, United Kingdom; 3 Department of Hematology, Hospital Clinic de Barcelona, Spain; 4 Department of Hematology, Radboud University Medical Center, Nijmegen, Netherlands; 5 Department of Hematology, Hospital Universitario Clinico San Carlos, Madrid, Spain; 6 Department of Hematology, Hospital Universitario Puerta de Hierro Majadahonda, Madrid, Spain; 7 Alexion Pharmaceuticals, Inc., Boston, MA, United States of America; 8 Department of Medicine, Jane Anne Nohl Division of Hematology, Keck School of Medicine of University of Southern California, Los Angeles, CA, United States of America; 9 Department of Medicine, Sunnybrook Health Sciences Centre, Toronto, ON, Canada; 10 Centre de Référence Aplasie Médullaire, Service d'Hématologie Greffe, Assistance Publique des Hôpitaux de Paris, Hôpital Saint-Louis, Paris, France; 11 Department of Hematology, West German Cancer Center, University Hospital Essen, University of Duisburg-Essen, Essen, Germany; 12 Department of Hematology, University Hospital Monklands, North Lanarkshire, Scotland; 13 Department of Haematology, St James' Institute of Oncology, Leeds, United Kingdom; Mayo Clinic, UNITED STATES

## Abstract

**Background:**

Eculizumab has transformed management of paroxysmal nocturnal hemoglobinuria (PNH) since its approval. However, its biweekly dosing regimen remains a high treatment burden. Ravulizumab administered every 8 weeks demonstrated noninferiority to eculizumab in two phase 3 trials. In regions where two PNH treatment options are available, it is important to consider patient preference.

**Objective:**

The aim of this study was to assess patient preference for ravulizumab or eculizumab.

**Methods:**

Study 302s (ALXN1210-PNH-302s) enrolled PNH patients who participated in the extension period of phase 3 study ALXN1210-PNH-302. In the parent study, eculizumab-experienced adult PNH patients received ravulizumab or eculizumab during a 26-week primary evaluation period. All patients in the extension period received ravulizumab. In study 302s, patient treatment preference was evaluated using an 11-item PNH-specific Patient Preference Questionnaire (PNH-PPQ^©^). Of 98 patients, 95 completed PNH-PPQ^©^ per protocol for analysis.

**Results:**

Overall, 93% of patients preferred ravulizumab whereas 7% of patients either had no preference (6%) or preferred eculizumab (1%) (*P* < 0.001). For specific aspects of treatment, ravulizumab was preferred (in comparison to no preference or eculizumab) on infusion frequency (98% vs. 0% vs. 2%), ability to plan activities (98% vs. 0% vs. 2%), and overall quality of life (88% vs. 11% vs. 1%), among other aspects. Most participants selected frequency of infusions as the most important factor determining preference (43%), followed by overall quality of life (23%).

**Conclusion:**

This study shows that a substantial proportion of patients preferred ravulizumab over eculizumab and provides an important patient perspective on PNH treatment when there is more than one treatment option.

## Introduction

Paroxysmal nocturnal hemoglobinuria (PNH) is a rare and potentially life-threatening hematologic disorder primarily caused by somatic mutations in the phosphatidylinositol glycan class A (*PIGA*) gene and is characterized by uncontrolled activation of the terminal complement pathway resulting in intravascular hemolysis [1]. The primary clinical manifestations of PNH are anemia, thrombosis, and smooth muscle dystonia [1]. Anemia is often caused by a combination of hemolysis and bone marrow failure. The major contributors to mortality and morbidity associated with PNH are thrombosis and renal complications [1, 2].

Eculizumab is a humanized monoclonal antibody against terminal complement protein component 5 (C5) that blocks terminal complement activation [1]. Eculizumab was the first approved treatment for patients with PNH [3, 4], and changed the paradigm of PNH management from supportive care to biologically targeted therapy, providing improvement in patient survival [5]. In phase 3 studies, eculizumab treatment decreased intravascular hemolysis, reduced need for blood transfusion, and improved PNH-related symptoms including fatigue [6, 7]. Although eculizumab is highly effective in the treatment of patients with PNH, 11–27% of eculizumab-treated patients may experience breakthrough hemolysis due to suboptimal C5 inhibition, infections, surgery, or pregnancy [8–10]. In addition, the biweekly dosing regimen for eculizumab remains a high treatment burden, has an impact on a patient’s health-related quality of life (HRQOL) [9, 11], and potentially influences treatment adherence.

Ravulizumab, the first long-acting complement inhibitor, which was recently approved for the treatment of PNH in the USA (December 2018), Japan (June 2019), Europe (July 2019), Canada (August 2019), and Brazil (September 2019), provides immediate, complete, and sustained inhibition of C5 with an 8-week dosing interval [8, 9, 12–14]. Two phase 3, multicenter, randomized, active-controlled, open-label studies (ALXN1210-PNH-301 and ALXN1210-PNH-302) demonstrated noninferior efficacy of ravulizumab relative to eculizumab with respect to the need for transfusions, normalizing lactate dehydrogenase, improving PNH-related fatigue, reducing the proportion of patients experiencing breakthrough hemolysis, and stabilizing hemoglobin levels in both C5 inhibitor-naive and C5 inhibitor-experienced-patients [8, 9]. The safety and tolerability of ravulizumab were also comparable to eculizumab [8, 9].

As patients with PNH now have two approved treatment options in a number of countries, it is important to consider patient preference when determining a treatment plan. The aim of this study was to assess patient preferences for ravulizumab (administered every 8 weeks [q8w]) or eculizumab (administered every 2 weeks [q2w]) in clinical trial substudy ALXN1210-PNH-302s, using an 11-item PNH-specific Patient Preference Questionnaire (PNH-PPQ^©^) [15].

## Materials and methods

### Study design

Study ALXN1210-PNH-302 (ClinicalTrials.gov identifier, NCT03056040, https://clinicaltrials.gov/ct2/show/NCT03056040), the parent 302 study, is an ongoing phase 3, open-label, randomized, active-controlled, multicenter study evaluating the safety and efficacy of ravulizumab versus eculizumab in adult patients with PNH who were clinically stable on eculizumab for at least 6 months prior to the start of the study. The study included a 26-week primary evaluation period in which patients received either the approved dose of eculizumab (900 mg, q2w) or weight-based dosing of ravulizumab (q8w) [8]. After completion of the 26-week primary evaluation period, all patients had the opportunity to enter an extension period (up to 3 years), during which those patients in the ravulizumab arm continued to receive ravulizumab (ravulizumab-ravulizumab) maintenance therapy; patients in the eculizumab arm were switched to ravulizumab (eculizumab-ravulizumab). Eculizumab-treated patients who switched to ravulizumab received a weight-based loading dose of ravulizumab followed 2 weeks later by weight-based maintenance doses q8w [8].

Substudy ALXN1210-PNH-302s (study 302s) was a non-interventional, noninvasive, non-randomized, multicenter study on a subset of patients enrolled in the extension period of study ALXN1210-PNH-302. It was planned to enroll at least 95 patients in study 302s and ended when the last patient completed the PNH-PPQ^©^. The protocol for study 302s was reviewed and approved by the Northwestern University Institutional Review Board.

### Patients

As previously reported [8], patients (≥18 years of age) with a diagnosis of PNH enrolled in the extension phase of the study had an option to participate in study 302s. Patients were included in study 302s when they had received a minimum of 2 ravulizumab maintenance doses during the extension period, and provided signed informed consent. There were no exclusion criteria for the study 302s. The PNH-PPQ^©^ was administered to the patients at a single time point.

### Paroxysmal Nocturnal Hemoglobinuria Patient Preference Questionnaire (PNH-PPQ^©^)

The 11-item PNH-PPQ^©^ has been previously described [15]. Briefly, it consists of the following questions: one question assessing overall treatment preference (Q1), nine questions (Q2a-i) evaluating treatment preference based on treatment characteristics (eg, “controlling fatigue,” “frequency of infusions”) with one question (Q2j) as a write-in option, one question (Q3) asking patients to indicate which treatment characteristic was most important for their overall medication preference, four questions evaluating aspects of treatment with eculizumab and four matching questions for ravulizumab (eg, “the frequency of infusions with Soliris [eculizumab] disrupted my life” and “the frequency of infusions with ALXN1210 [ravulizumab] disrupted my life;” Q4-11). For Q1, “eculizumab” and “ravulizumab” were each coded as 1 and “I do not have a preference” was coded as 2. For Q2a-j, “strongly prefer eculizumab or somewhat prefer eculizumab” was coded as 0, “I do not have a preference” was coded as 1, and “somewhat prefer ravulizumab or strongly prefer ravulizumab” was coded as 2. All questions were scored as described in Table 1.

**Table 1:** Summary of Paroxysmal Nocturnal Hemoglobinuria Patient Preference Questionnaire^©^ content* [15].

Question (Q) Item	Response/Score
Q1. Overall preference question, which asked patients to indicate which of the 2 medications they prefer based on their experience with the 2 treatments	0 = eculizumab
	1 = ravulizumab
	2 = I do not have a preference
Q2a-2j. Questions evaluating preference for eculizumab or ravulizumab based on controlling fatigue (a), controlling symptoms other than fatigue (b), frequency of infusions (c), side effects of treatment (d), convenience of receiving treatment (e), being able to plan activities (f), effectiveness of the medication until the next infusion (g), anxiety related to the infusion (h), and your overall quality (i)	A 5-point ordered response scale ranging from 0 to 4
	0 = strongly prefer eculizumab
	1 = somewhat prefer eculizumab
	2 = I do not have a preference
	3 = somewhat prefer ravulizumab
And one write-in option (j)	4 = strongly prefer ravulizumab
Q3. One question asking patients to indicate which treatment factor was most important for their overall medication preference	Choice of a through j from Q2
Q4 through Q7. Four questions evaluating aspects of treatment with eculizumab	A 5-point scale ranging from 0 to 4
	0 = Not at all
	1 = A little bit
	2 = Some-what
	3 = Quite a bit
	4 = Very much
Q8 through Q11. Four questions evaluating those same aspects of treatment with ravulizumab	A 5-point scale ranging from 0 to 4
	0 = Not at all
	1 = A little bit
	2 = Some-what
	3 = Quite a bit
	4 = Very much

*The questions listed in this table are rephrased summaries adapted from the actual PNH-PPQ [15]. Critical elements needed for a valid assessment of patient preference are missing from this summary; for more information on the full PNH-PPQ, please review the publication by Kaiser K et al. Patient Prefer Adherence. 2020;14:705–715. For any questions or copyright information, please contact Alexion Pharmaceuticals, Inc.

### Statistical analysis

Sample size determinations were informed by statistical power analyses to detect the hypothesized proportion of patients reporting a preference for treatment with ravulizumab rather than eculizumab or having no preference based on Q1. Under the null hypothesis that 50% of patients prefer ravulizumab, a sample size of 95 patients would have at least 80% power to detect an observed proportion of 65% or greater using a 2-sided exact binomial test with a Type I error of 0.05. Statistical analyses were performed for each item in the PNH-PPQ^©^, and for the purpose of analysis, the evaluable sample was defined as all patients who completed Q1 of the PNH-PPQ^©^. All statistical analysis used the evaluable sample.

For Q1, although the responses for preference for “eculizumab” and “I do not have a preference” were captured separately, for the purpose of this analysis those responses were collapsed. For Q2a-Q2i, proportions and frequencies of patients preferring ravulizumab, eculizumab, and those who had no preference were calculated. For Q3, the frequency of patients choosing each treatment characteristic as most important for determining treatment preference was calculated. For Q1 through Q3, if the 95% confidence interval (CI) did not include zero, the comparison was considered to be statistically significant. For Q4–11, mean responses to matching questions (Q4 vs. Q8; Q5 vs. Q9; Q6 vs. Q10; Q7 vs. Q11) were compared with paired *t* tests and standardized effect sizes (*d*). Paired *t* tests were calculated to compare means on complementary questions within patients. If responses were not always normally distributed, *P* values were calculated using Wilcoxon signed-rank tests in sensitivity analyses; this has been footnoted when done so. Standardized effect sizes were calculated as mean of the differences between ravulizumab and eculizumab scores divided by the standard deviation of the differences. Effect sizes (absolute value) were classified as small (0.20 to <0.50), medium (0.50 to <0.80), or large (≥0.80) in magnitude [16]. Continuous variables were summarized using descriptive statistics, including number of observations and mean, standard deviation (SD), median, minimum, and maximum values. Categorical variables were summarized by frequency counts and percentages of patients. All tests were performed in SAS v9.4. and *P* < 0.05 was considered statistically significant.

## Results

### Patient characteristics

Of 98 patients enrolled in the study 302s, 3 patients did not respond to Q1 of the PNH-PPQ^©^ and were excluded from the analysis. The remaining 95 patients came from 8 countries (Great Britain, Spain, USA, Canada, Germany, Netherlands, France, and Australia). Among those patients, approximately 56% were male and 44% were female (Table 2). The largest numbers of patients were recruited from Great Britain (37%) and Spain (20%). On average, participating patients were 50 years old (range: 22–78 years), had been diagnosed with PNH 14 years earlier (range: 2–48 years), and had received eculizumab treatment for 6 years (range: 1–16 year) prior to study. In addition, the mean time between the last randomized study treatment in the primary evaluation period and completion of the PNH-PPQ^©^ was 306 days (range: 196–457 days). Overall, patient characteristics were comparable between both treatment arms.

**Table 2:** Characteristics of the study population.

	Total (N = 95)	Ravulizumab Primary (n = 50)	Switched from Eculizumab to Ravulizumab (n = 45)
**Country, n (%)**			
Great Britain	35 (37)	20 (40)	15 (33)
Spain	19 (20)	10 (20)	9 (20)
USA	9 (9)	3 (6)	6 (13)
Canada	8 (8)	5 (10)	3 (7)
Germany	8 (8)	1 (2)	7 (16)
Netherlands	8 (8)	5 (10)	3 (7)
France	5 (5)	4 (8)	1 (2)
Australia	3 (3)	2 (4)	1 (2)
**Age,** **mean years (SD, range)**	50 (13, 22–78)	48 (13, 22–78)	52 (13, 25–73)
**Sex, female n (%)**	42 (44)	22 (44)	20 (44)
**Years since diagnosis, mean (SD, range)**	14 (10, 2–48)	14 (10, 3–39)	13 (11, 2–48)
**Years on eculizumab before study, mean (SD, range)**	6 (4, 1–16)	6 (3, 1–15)	6 (4, 1–16)
**Days between last randomized study treatment and PNH-PPQ**^**©**^**, mean (SD, range)**	306 (55, 196–457)	331 (54, 236–457)	279 (42, 196–420)
**History of major adverse vascular events, n (%)**	24 (25)	14 (28)	10 (22)

PNH-PPQ^©^, Paroxysmal Nocturnal Hemoglobinuria-specific Patient Preference Questionnaire; SD, standard deviation.

### Overall treatment preference

**Fig 1:**
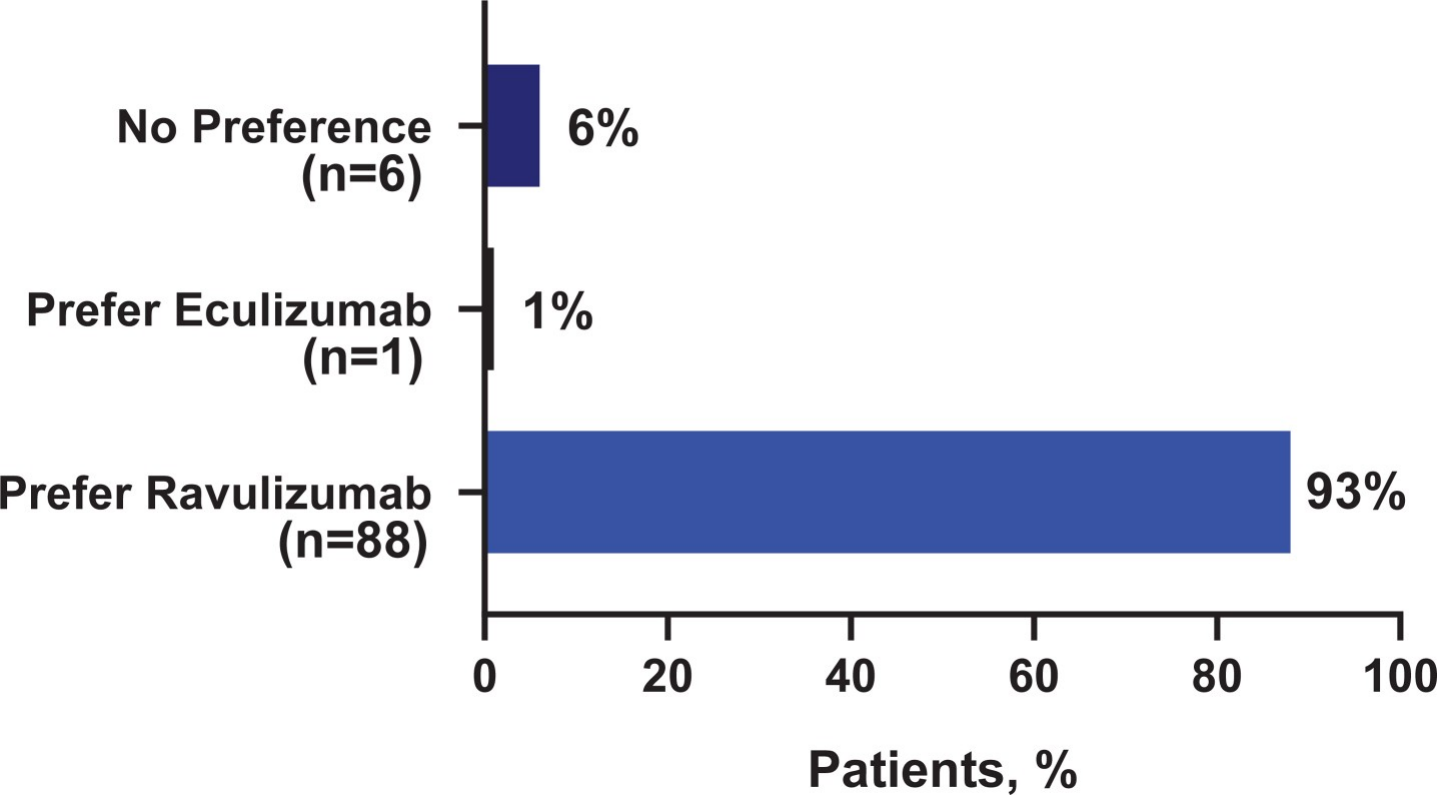
Overall treatment preference (N = 95).

Of the total evaluable patients (n = 95), 93% (n = 88) indicated an overall preference for ravulizumab, 1% (n = 1) reported an overall preference for eculizumab, and 6% (n = 6) reported “no preference.” The proportion of patients reporting a preference for ravulizumab (93%, 95% confidence interval [CI]: 87%;98%) was significantly higher as compared to patients who reported either a preference for eculizumab or no preference (7%, 95% CI: 2%;12%) (Fig 1).

### Factors determining treatment preference

**Fig 2:**
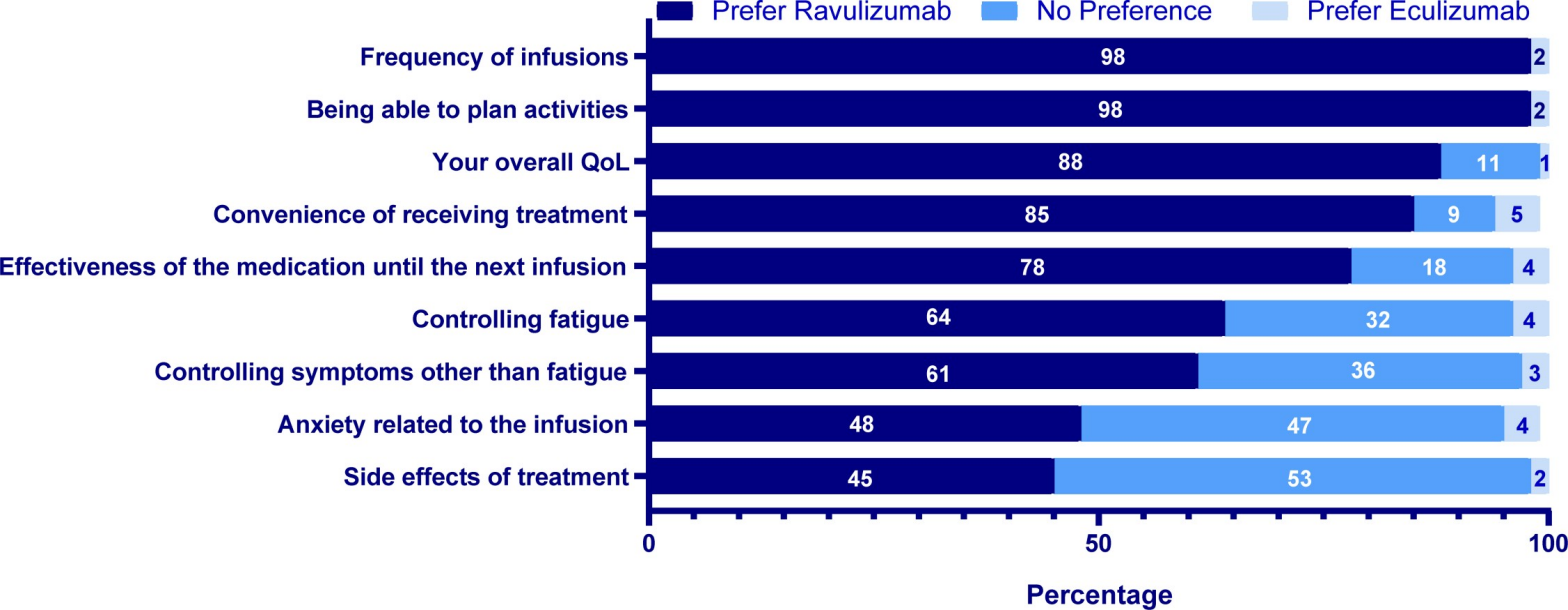
Factors driving patients’ treatment preference^a,b^ (N = 95). ^a^Preference response was defined as responding “Strongly” or “Somewhat” prefer respective drug. ^b^1 missing response for “controlling symptoms other than fatigue” and “being able to plan activities” and “effectiveness of the medication until the next infusion; 2 missing responses for “your overall quality of life”.

In general, ravulizumab was widely preferred as compared with no preference or eculizumab for frequency of infusions (98% vs. 0% vs. 2%), ability to plan activities (98% vs. 0% vs. 2%), overall HRQOL (88% vs. 11% vs. 1%), convenience of receiving treatment (85% vs. 9% vs. 5%), and effectiveness of medication until the next infusion (78% vs. 18% vs. 4%). With respect to treatment characteristics related to side effects of treatment and anxiety related to infusions, 45% (vs. 53% vs. 2%) and 48% (vs. 47% vs. 4%) of patients, respectively, preferred ravulizumab compared with no preference or eculizumab. In contrast, fewer patients preferred eculizumab over ravulizumab across all factors (Fig 2).

### Patients’ single most important treatment factor for deciding medication preference

**Fig 3:**
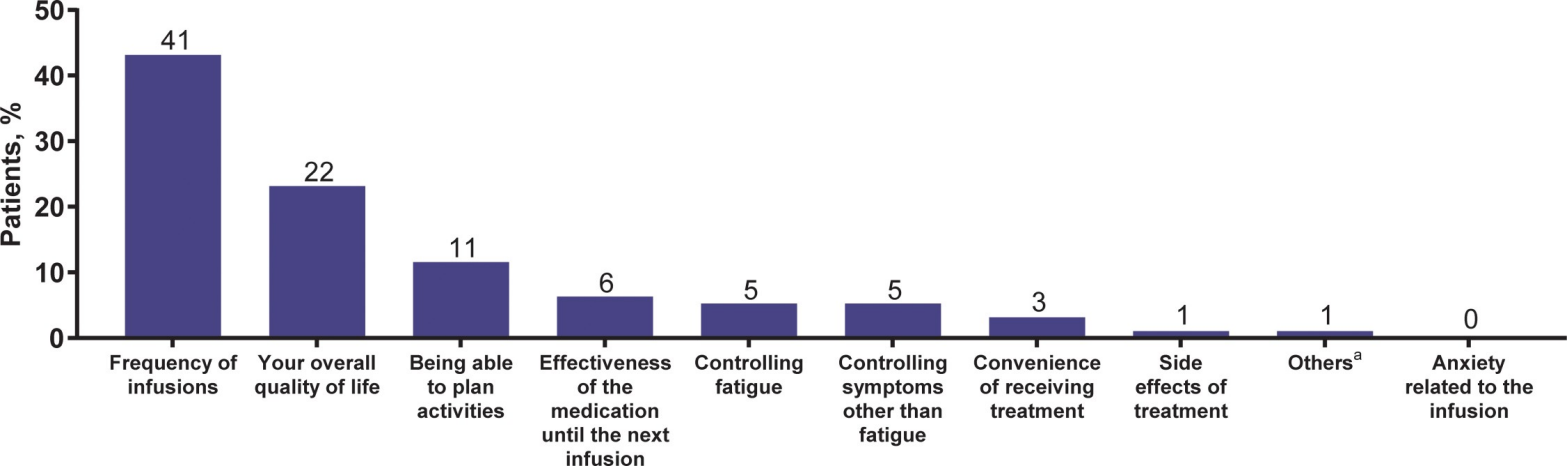
Patients’ most important factor for deciding medication preference (N = 95). The number of patients selecting each preference is at top of bar. ^a^Participants selecting “Other” were prompted to provide details.

Frequency of infusions was selected by the majority of patients (43%; n = 41) as the most important determinant of treatment preference, followed by overall HRQOL (23%; n = 22) and being able to plan activities (12%; n = 11) (Fig 3).

### Impact of treatment on patient HRQOL

For the treatment-related factors that were positively stated (“effective in treating symptoms of PNH” and “while I was receiving treatments, I was able to enjoy life”), patients rated ravulizumab higher than eculizumab (Table 3). In contrast, patients rated ravulizumab lower versus eculizumab on the treatment-related aspects that were negatively stated (“the frequency of infusions disrupted my life” and “after receiving infusions, I had fatigue”) (Table 3). All of these comparisons favored ravulizumab. In terms of standardized effect sizes, the factors “the frequency of infusions disrupted my life” and “while I was receiving treatments, I was able to enjoy life” showed large magnitude effect sizes (1.46 and 0.88, respectively), whereas the standardized effect size for “after receiving infusions, I had fatigue” was medium magnitude (0.56).

**Table 3:** Impact of treatment on measures of patient health-related quality of life.

	Ravulizumab^a^	Eculizumab^a^	Mean of Differences (ravulizumab-eculizumab)	SD	Effect Size^b^	*P* Value^c^
**The frequency of infusions disrupted my life**	0.39	2.21	−1.82	1.24	1.46	<0.001
**After receiving infusions, I had fatigue**	0.62	1.21	−0.59	1.05	0.56	<0.001
**Effective in treating symptoms of PNH**	3.59	3.36	0.23	0.87	0.27	0.01
**While I was receiving treatments, I was able to enjoy life**	3.62	2.81	0.81	0.93	0.88	<0.001

PNH, paroxysmal nocturnal hemoglobinuria; QoL, quality of life; SD, standard deviation.

^a^Mean of responses on an agreement scale of 0 = “Not at all” to 4 = “Very much.” Higher means indicate greater agreement. For this reason, lower scores on negatively worded questions are more favorable and higher scores on positively worded questions are more favorable.

^b^Effect sizes are calculated as the difference in mean scores divided by the standard deviation of the mean differences. Normative standards for effect sizes (absolute) are: small, 0.20 to <0.50; medium, 0.50 to <0.80; large ≥0.80.

^*c*^*P* value from paired *t* test; *P* values calculated using the Wilcoxon signed-rank test gave similar findings except for “Effective in treating symptoms of PNH” for which the Wilcoxon test gave a *P* = 0.003.

## Discussion

This study demonstrated that most patients (93%) with PNH preferred ravulizumab compared to eculizumab; and this was statistically significant (95% CI: 87%; 98%). Ravulizumab was preferred based on multiple factors including infusion frequency (q8w vs. q2w), ability to plan activities, overall HRQOL, convenience of treatment, and effectiveness of medication until the next infusion. Among these factors, infusion frequency was rated the most important for deciding medication preference in patients with PNH, despite the duration of a single treatment with ravulizumab being longer than the duration of a single treatment with eculizumab (ie, 3 hours vs. 35 minutes). Furthermore, negative factors relating to HRQOL also played a role in determining patient treatment preference. This study’s results highlight the importance of these key factors in patient determination of treatment preference for PNH.

In addition, patients preferred ravulizumab over eculizumab due to “effectiveness until next infusion,” “controlling fatigue,” and “controlling symptoms other than fatigue.” These results suggest that patients may have preferred ravulizumab because it positively influences HRQOL and decreases PNH-related symptoms for longer duration (up to 8 weeks) relative to eculizumab. Consistent with this, patients rated ravulizumab more highly than eculizumab in terms of “effective in treating symptoms of PNH” and “while I was receiving treatments, I was able to enjoy life,” further indicating that ravulizumab is viewed positively by patients with PNH. Furthermore, as patients feel they are better able to plan their lives, they may become more active contributors to society as well as achieve an increased sense of normalcy. For patients who had no preference for treatment, one of the factors that may have influenced their decision was no differences in side effects of treatment. Similarly, for the one patient who preferred eculizumab over ravulizumab, “convenience of receiving treatment” was one of the factors that may have contributed to their treatment preference and may relate to the shorter infusion time needed with eculizumab [3] treatment compared with ravulizumab [17]. However, both these factors, side effects of treatment and convenience of receiving treatment, were not the leading factors for deciding treatment preference among all patients (see Fig 3).

In other areas of therapy including type 2 diabetes (T2D) and osteoporosis [18], patients have been shown to prefer less frequent dosing over more frequent dosing. Patients with T2D preferred weekly injection over daily injection based on patient-reported outcome (PRO) measures [19]. In a study evaluating preferences for preventative osteoporosis drug treatment, participants reported that a pill once monthly was preferred over a pill once weekly or other route of drug administration such as monthly and weekly injection [20]. Although the literature generally highlights that most patients prefer less frequent dosing when possible, important elements such as mode of administration, location of treatment (home or clinic), convenience, and lifestyle can influence treatment and dosing preferences. Some patients may prefer more frequent dosing because it fosters close interaction with health care professionals, which can minimize their anxiety related to potential long-term ineffectiveness of a drug and serious consequences associated with the ineffectiveness. Thus, in the context of PNH, this study helps in understanding and improving patient treatment experience.

Most PRO measures and evaluations of drug treatment focus on traditional HRQOL or symptom outcomes such as the European Organisation for Research and Treatment of Cancer Quality of Life Questionnaire (EORTC QLQ)-C30 and the Functional Assessment of Chronic Illness Therapy Fatigue Instrument (FACIT-Fatigue). Although the EORTC QLQ-C30 and the FACIT-Fatigue have been administered to patients with PNH in clinical trials [11] and in the international PNH Registry[21] to assess overall HRQOL following PNH treatments (eculizumab, stem cell transplantation, immunosuppressants), these instruments do not capture patient preferences related to treatment [11].

The PNH-PPQ^©^ was developed using a rigorous process consistent with Food and Drug Administration guidelines for the development of PRO measures [22] to capture patients’ overall treatment preference and their preference based on key aspects of PNH treatment including symptom management, infusion frequency, and overall burden of treatment [15]. The National Institute for Health and Care Excellence (NICE) guidelines [23] also emphasize the importance of understanding patient preferences and needs for supporting patient decision making. The 11-item PNH-PPQ^©^ provides a patient-centered approach for evaluating preferences specifically for the treatment of PNH and assesses patient preference between two treatments as well as the factors influencing those preferences.

Although the demographics of the 302s study population are similar to the characteristics of the general population of PNH patients, several limitations should be considered when interpreting the results of this study. First, the PNH patients who participated in study 302s were part of the parent 302 study and were treated per study protocol regardless of their treatment preference. The PNH-PPQ^©^ was administered in the extension phase of the 302 study, and patients were required to have had at least two administrations of ravulizumab to ensure sufficient experience to respond to the PNH-PPQ^©^. Therefore, patient responses to the questionnaire were obtained irrespective of their treatment preference. However, patients in the 302 substudy had to agree to participate in the 302 extension study and might have had a predisposition to prefer ravulizumab because of less frequent infusion, representing a potential bias. Nevertheless, to assess patient treatment preference, patients experienced with the treatments in question are needed. Second, the PNH-PPQ^©^ was administered to patients with PNH who participated in the study 302s to gain additional insights into their experiences with ravulizumab and eculizumab and their treatment preferences. However, the amount of time between patients’ last randomized treatment and completion of the PNH-PPQ^©^ presents potential bias in time-based recall of their experiences. Therefore, the patient preference results should be confirmed in real-world analyses to reduce the potential limitation. Finally, despite rationale for a strong hypothesis that most patients would prefer the 8-week regimen over the 2-week regimen, this was not a foregone conclusion. It was possible that patients would worry about changing regimens or would prefer to stay with the treatment they were used to (recall that mean eculizumab treatment experience prior to study entry was 6 years), had known efficacy and safety, and had been approved by regulatory agencies for several years. Indeed, given that data sets on patients with PNH are rare, especially those assessing treatment preference, this data set represents a unique opportunity to test the hypothesis because it includes a rather large number of patients with PNH who have experience with both eculizumab and ravulizumab.

In conclusion, the results from the PNH-PPQ^©^ in study 302s showed that a majority of patients with PNH preferred ravulizumab over eculizumab. Ravulizumab was preferred because of reduced infusion frequency (q8w vs. q2w), better ability to plan activities, improved overall HRQOL, more convenient treatment, and effectiveness of the medication until the next infusion. These results clearly demonstrate that patients with PNH deliberately consider treatment frequency and its impact on their lives when evaluating treatment preference. Overall, these findings provide an important patient perspective on treatment preferences for PNH when there is more than one treatment option.

## Supporting information

S1 File (DOCX)
