# Supplementary material for: Patient preferences and quality of life implications of ravulizumab (every 8 weeks) and eculizumab (every 2 weeks) for the treatment of paroxysmal nocturnal hemoglobinuria
Source: PLoS One. 2020 Sep 4;15(9):e0237497. doi: 10.1371/journal.pone.0237497 (PMC7473546; doi:10.1371/journal.pone.0237497)
Supplement: S1 File — (DOCX) [file pone.0237497.s001.docx]

**Patient Preferences and Quality of Life Implications of Ravulizumab (every 8 weeks) and Eculizumab (every 2 weeks) for the Treatment of Paroxysmal Nocturnal Hemoglobinuria**

**Background**

PNH is a rare, potentially life-threatening, **and usually life-long blood disease**. The red blood cells of patients with PNH lack important proteins involved in **immune system regulation,** which leads to red blood cell destruction, a phenomenon known as **hemolysis**.

Hemolysis is responsible for many PNH symptoms including fatigue, stomach pain, trouble swallowing (dysphagia), and dark colored urine (hemoglobinuria), and erectile dysfunction. It also causes **serious health problems** such as blood clots (potentially leading to stroke or heart attack), kidney disease, and/or damage to your other organs. There may be major health problems with PNH that can be life-threatening.

There are treatments that have been developed to manage PNH, aiming to reduce hemolysis by blocking the part of the immune system that destroys red blood cells in patients with PNH.

| **ECULIZUMAB**  **is approved and is used as the standard to treat people with PNH. It is a lifelong treatment, which is given to people with PNH every 2 weeks through in intravenous (into the vein) infusion.** | **RAVULIZUMAB**  **is a newly approved treatment for people with PNH. It is also a lifelong treatment, which is given every 8 weeks through an intravenous infusion.** |
| --- | --- |

In two clinical studies, ravulizumab was found to be as safe and effective, or no worse than eculizumab at treating PNH. Since two treatments are now available to patients with PNH, it is important to study which is preferred by patients to improve their quality of life and make it easier to take their treatments over time.


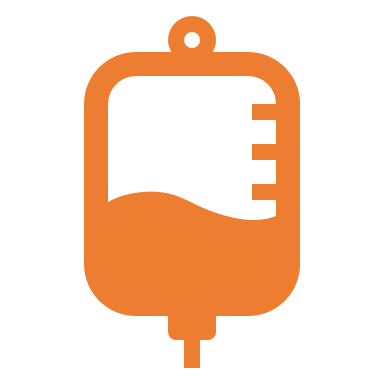

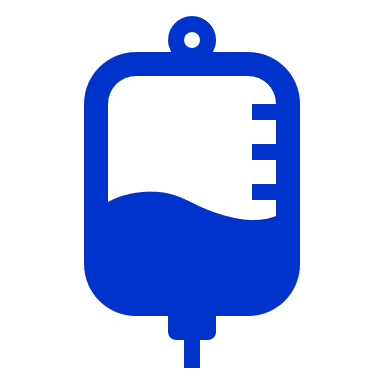


**Ravulizumab**

Administered every **8 weeks**

**Eculizumab**

Administered every **2 weeks**


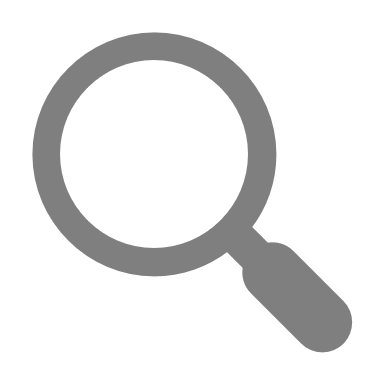


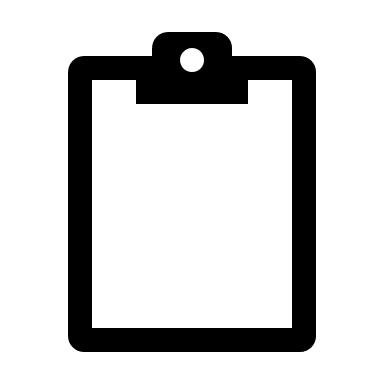


**Study information**

This study assessed **patient preference** for ravulizumab or eculizumab using a **PNH Patient Preference Questionnaire** (PNH-PPQ^©^)^*^. The survey included questions about **overall treatment preference**, and preferences regarding specific factors such as treatment **infusion frequency**, the ability to **plan activities**, and **quality of life**.

*Summary of PNH-PPQ^©^ is included as Table 1 in the manuscript.

In total**, 95 adult patients** from **8 countries**, who had experience taking both treatments, completed the questionnaire. Patients included in this study received eculizumab for at least 6 months and ravulizumab for at least 2 months.

**Study results**

Overall, **93% of patients preferred ravulizumab**, while 6% did not have a preference, and 1% preferred eculizumab.

When considering the reason for treatment preference, ravulizumab was preferred in the majority of cases

- **Infusion frequency**: **98% of patients preferred ravulizumab** (8-week interval) and 2% preferred eculizumab (2-week interval)
- **Ability to plan activities** (without treatment interference): **98% of patients preferred ravulizumab** and 2% preferred eculizumab
- **Improvement in quality of life**: **88% of patients preferred ravulizumab**, 11% did not have a preference, and 1% preferred eculizumab
- **Side effects of treatment: 45% of patients preferred ravulizumab** and 2% preferred eculizumab


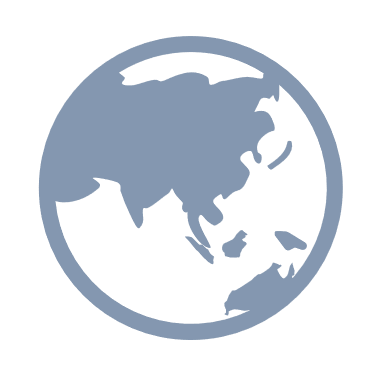

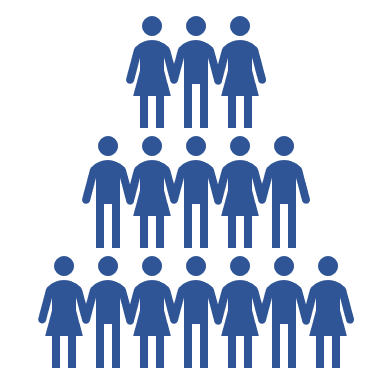

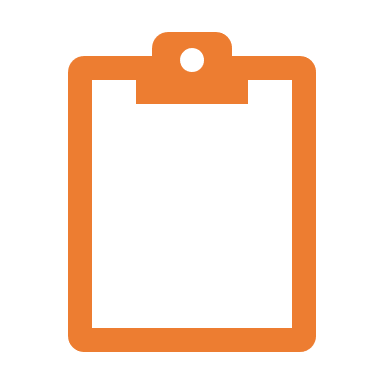


Among patients who had experience taking both eculizumab and ravulizumab, frequency of infusions was reported as the most important factor in determining treatment preference (n=41), followed by overall quality of life (n=22).

QoL, Quality of life.

^a^Respondents selecting “Other” were prompted to provide details.

**What do these findings mean for patients?**

This study shows that the **majority (93%) of patients preferred treatment with ravulizumab** over eculizumab. It also provides important information on **patient preferences** for PNH treatment schedules and their impact on quality of life.

**Who sponsored this study?**

Alexion Pharmaceuticals, Inc. (Boston, MA, USA)

Alexion would like to thank all the patients, physicians, and patient organizations for their research efforts.

**This summary is based on the following research article:**

Peipert JD, Kulasekararaj AG, Gaya A, et al. Patient preferences and quality of life implications of ravulizumab (every 8 weeks) and eculizumab (every 2 weeks) for the treatment of paroxysmal nocturnal hemoglobinuria.
